# The microbiome of the upper airways: focus on chronic rhinosinusitis

**DOI:** 10.1186/s40413-014-0048-6

**Published:** 2015-01-27

**Authors:** Thanit Chalermwatanachai, Leydi Carolina Velásquez, Claus Bachert

**Affiliations:** Department of Oto-Rhino-Laryngology, The Upper Airways Research Laboratory (URL), Ghent University Hospital, Ghent, 9000 Belgium; Department of Otolaryngology, Phramongkutklao Hospital and College of Medicine, Royal Thai Army, Bangkok, 10400 Thailand; Basic Biomedical Sciences Department, Health Faculty, Universidad Industrial de Santander, Bucaramanga, Colombia; Division of ENT Diseases, Clintec, Karolinska Institutet, Stockholm, Sweden

## Abstract

Upper airway diseases including allergic rhinitis, chronic rhinosinusitis with or without polyps, and cystic fibrosis are characterized by substantially different inflammatory profiles. Traditionally, studies on the association of specific bacterial patterns with inflammatory profiles of diseases had been dependent on bacterial culturing. In the past 30 years, molecular biology methods have allowed bacterial culture free studies of microbial communities, revealing microbiota much more diverse than previously recognized including those found in the upper airway.

At presence, the study of the pathophysiology of upper airway diseases is necessary to establish the relationship between the microbiome and inflammatory patterns to find their clinical reflections and also their possible causal relationships. Such investigations may elucidate the path to therapeutic approaches in correcting an imbalanced microbiome.

In the review we summarized techniques used and the current knowledge on the microbiome of upper airway diseases, the limitations and pitfalls, and identified areas of interest for further research.

**Electronic supplementary material:**

The online version of this article (doi:10.1186/s40413-014-0048-6) contains supplementary material, which is available to authorized users.

## Introduction

It is generally believed that exposure to microorganism compromises health. Reduced exposure to microbiota results in decrease of incidence of infectious diseases but may adversely increase the incidence of allergic disorders [1-3]. Recent developments of culture-independent tools make it possible to identify microbial species not previously detected by conventional methods. Unbeknownst to us, man had been living with these microoraganisms since the dawn of time.

The human body harbors from 10 to 100 trillion microbes which greatly outnumber our own human cells [4]. This bacterial assemblage has been coined, “the human microbiota” [4]. Subsequently, a project called “Human Microbiome” was established to investigate the flora in healthy volunteers and their relationship to human health and disease [5]. The study of the host-microbe relationship has shown that microbes play a major role in our well-being [4,6]. Alterations of microbial composition have been linked to several human diseases [4]. There is also evidence showing that, in the respiratory system, composition of airway microbiota varies between healthy people and people with diseases such as asthma [6-8] and cystic fibrosis (CF) [8,9]. Unfortunately, with limited studies currently available, it cannot be concluded with the same degree for chronic rhinosinusitis (CRS) [10].

Research on microbiome in CRS is therefore needed to elucidate pathophysiology of this disease such as; 1) the relationship between the microbiome and inflammatory patterns, 2) possible causal relationships between microbe and CRS, 3) investigation of the microbiome regarding possible therapeutic properties. Dysregulation of the interactions between the immune system and commensal bacteria is a contributing factor to the development and chronicity of a number of inflammatory diseases [11]. Microorganisms in the gut may play a significant role in regulating T helper cells (Th cells), regulatory T cells (Tregs) and dendritic cells as well as Toll-like receptor expression in sentinel cell (macrophage and dendritic cells) which are relevant to airway illnesses such as asthma and allergic diseases [10].

### Techniques in microbiota study

Principal approaches to analyze human microbiota are: culture-dependent and culture-independent techniques. Culture-dependent methods involve isolation and culturing of microorganisms prior to their identification according to morphological, biochemical or genetic characteristics. These methods are time-consuming, due to culture and bias, as certain media and growth conditions favor the growth of some bacteria over others [12]. In addition, this approach may not provide a true reflection of the diversity of microbes in a sample. A “no growth” result does not necessarily imply that a sample is sterile. It is estimated that up to 99% of microorganisms observable in nature typically cannot be cultured by standard techniques [13]. Un-cultivability is a widespread condition that includes: (i) organisms for which the specific growth requirements (nutritional, temperature, aeration, etc.) are not fulfilled; (ii) slow-growing organisms are out-competed in the presence of fast-growing microorganisms and (iii) disfavored organisms, which cannot stand the stressful conditions imposed by cultivation [13,14]. This approach camouflages the true bacterial community. There needed to be a better approach to analyze these microorganisms.

Since the 1980s, the application of molecular detection methods has allowed culture-independent investigations of the microbial communities [15]. Molecular techniques have proven effective in characterizing complex microbial assemblages in environmental samples [16]. However, an important usefulness of molecular techniques is the ability to detect genetic materials of non-viable microorganisms [17,18]. Culture-independent methods are based on the direct analysis of bacterial DNA (or RNA) without culturing. Due to the sensitivity of these techniques, special care and attention is required for procedures that include sample collection and handling, DNA extraction, amplification of gene fragments, distinction of different fragments, identification of microorganism and analysis of the microbial community [15].

For bacterial identification, the predominant gene target for amplification has been the 16S ribosomal RNA gene (or 16S rRNA) [19,20], which is a component of the 30S small subunit of prokaryotic ribosomes [21]. It has been widely targeted because of (i) its presence in almost all bacteria, often existing as a multigene family, or as operon; (ii) the conservation of the 16S rRNA gene, suggesting that random sequence changes are a measure of time (evolution) rather than a reflection of different bacteria; and (iii) the size of 16S rRNA genes (1,500 bp) being large enough for informatic purposes [22]. Moreover, there are several available reference sequences and taxonomies databases such as Greengenes, SILVA and the Ribosomal database project [23]. However, amplification of target genes using polymerase chain reaction (PCR) has made it impossible to completely avoid PCR-based biases and chimera production. It thus may distort the level of diversity and bacterial composition in a sample because of the amplification of pseudogenes [24]. Therefore, other technologies are often used as complementary approaches to 16S rRNA gene sequencing for reducing distortion of bacterial diversity and composition. They are DNA microarray, fluorescence in situ hybridization (FISH), and quantitative PCR (qPCR), and are based on oligonucleotide probes and primers that target the ribosomal RNA sequences or other genes in different hybridization procedures. Thus these techniques require a prior knowledge of the microbial DNA sequence. A DNA microarray (also commonly known as DNA chip or biochip) is a collection of microscopic DNA spots (oligonucleotide probes) attached to a solid surface. It is usually used for gene expression analysis or screening of single nucleotide polymorphisms. The FISH technique uses fluorescent probes that bind to only those parts of the chromosome with which they show a high degree of sequence complementarity. It detects and localizes the presence of specific DNA sequences on chromosomes. qPCR or real-time PCR follows the general principle of PCR with the advantage of detecting the amount of initial DNA in samples using either fluorescent DNA-binding dyes or fluorescence-tagged oligonucleotide probes [15].

The introduction of next generation sequencing changed the history of genomic research as it increased sequencing throughput, and did not require prior cloning steps [25]. These technologies are not only changing our genome sequencing approaches and the associated timelines and costs [26], but also developing many exciting fields such as metagenomics, metatranscriptomics and single-cell genomics [15]. Three platforms for high throughput parallel DNA sequencing are in reasonably widespread use at present: the Roche/454 FLX, the Illumina (MiSeq, HiSeq, and NextSeq), and the Ion Torrent.

At presence, researchers have a large choice in formulating methodological strategies: depending on the access to the technology, budget, and objectives of research. Each culture-independent methodology has its own limitations and biases, investigators must take additional measures; for example one may use more than one molecular technique or a culture-dependent approach in parallel to provide additional validation of results and reduce the possibility of false findings due to methodological errors and biases. Although the culture-independent techniques have the ability to detect more microbes than culture technique, the culture-dependent methods so far remain a better means of obtaining individual isolates contributing and obtaining isolates for further assays.

### Microbiota in allergic rhinitis

As the gate into our body, the respiratory tract itself harbors a heterogeneous microbiota that decreases in biomass from upper to lower tract [27]. Even in health, recent findings indicated that direct exposure to bacterial communities in the airways may provide an explanation for how commensal bacteria can regulate chronic airway inflammation [11]. Since the observation that infections within households in early childhood have a role in preventing allergic rhinitis [3], numerous epidemiologic and experimental studies have sought to clarify and extend the so-called hygiene hypothesis with respect to asthma and other allergic and autoimmune disorders. The evidence supporting the hygiene hypothesis established the “microbiota (microflora) hypothesis”. This concept proposes that perturbations in gastrointestinal bacteria have disrupted the mechanisms of mucosal immunologic tolerance, which has led to an increase in the incidence of allergic airway disease [28]. Independent studies found that a reduced diversity of the gut microbiota in infancy is associated with an increased risk of allergic manifestation at school age [29-31]. The association between the composition of microbiota in the intestine, asthma and allergic disease is nowadays of high interest [32,33], although the exact mechanism of the interaction of intestinal-systemic immunity is still not defined [34,35]. There are several publications reviewing the relationships between intestinal microbes and asthma [7,10,36]. Suggesting that the intestinal microbiome contributes to the regulation of local and systemic inflammatory responses via short-chain fatty acids, a product of fermentation of dietary fibers by intestinal bacteria [11]. Following this model, it is likely that the respiratory microbiota may also have an impact on airway inflammation in allergic responses [1,11], however, this needs further investigation.

### Microorganisms in the airways of cystic fibrosis patients

Cystic fibrosis (CF) is an autosomal recessive genetic disorder that affects among other organs the lungs and sinuses. It is characterized by abnormal transport of chloride and sodium across the epithelium, leading to thick, viscous secretions. This leads to defective mucociliary clearance and chronic airway infection with a complex microbiota [37]. Lung disease in cystic fibrosis results from chronic airway infection and inflammation leading to progressive bronchiectasis and respiratory failure [38]. Specimens for molecular microbial analysis in CF have for the most part all derived from sputum [37,39-42], while swabs from the middle meatus in patients with sinusitis were not used until now. Thus, no conclusions can be made for the upper airways yet. Samples consisted of serial collections of more than six patients in most of the studies [37,42-44].

Previous studies indicated that exacerbations might be associated with changes in microbial densities and the acquisition of new microbial species [37]. Bacterial pathogens, including *Pseudomonas aeruginosa*, *Staphylococcus aureus*, and *Burkholderia cepacia* are known contributors to such exacerbations. Recent studies using the latest culture-dependent techniques and culture-independent molecular techniques have broadened our view of CF airway bacterial communities [38]. Each CF patient presented a unique microbiome [40]. The species present tended to vary more “between” than “within” subjects, suggesting that each CF airway infection is unique, with relatively stable and resilient bacterial communities [44]. The diversity and species richness of fungal and bacterial communities were significantly lower in patients with decreased lung function and poor clinical status [39]. The authors observed a strong positive correlation between low species richness and poor lung function [37]. These findings show the critical relationship between airway bacterial community structure, disease stage, and clinical state at the time of sample collection [42].

The main microorganisms found in CF airways are the genera *Haemophilus*, *Pseudomonas, Staphylococcus* and *Stenotrophomonas*. Less common are gram-negatives, *Streptococcus* and *Mycobacterium spp* [45]. Most bacteria of CF airways are difficult to culture using conventional clinical methods; therefore, molecular approaches may confirm or reveal novel bacteria that might be related to the pathogenesis of cystic fibrosis. Examples of interest are the *Streptococcus milleri* group (*Streptococcus anginosus*, *Streptococcus intermedius, Streptococcus constellatus*) [46], *Pseudomonas intermedia* [46], and *Gemella species* [47] (Table 1). Further experiments suggested that these bacteria could act as co-pathogens or enhance the virulence of conventional CF pathogens [48].

**Table 1:** **Summary of cystic fibrosis microbiota studies; type of sample, technique used and genus identified**

**Author**	**Year**	***n***	**Sample**	**Techinque**	***Achromobacter***	***Actinomyces***	***Atopobium***	***Bacteroidetes***	***Burkholderia***	***Campylobacter***	***Capnocytophaga***	***Craurococcus***	***Fusobacterium***	***Granulicatella***	***Haemophilus***	***Lactobacillus***	***Leptotrichia***	***Ochrobactrum***	***Peptostreptococcus***	***Porphyromonas***	***Prevotella***	***Pseudomonas***	***Rhizobium***	***Rothia***	***Staphylococcus***	***Stenotrophomonas***	***Streptococcus***	***Veillonella***	**Ref.**
Salipante SJ *	2013	60 CF	Sputum	16 s rRNA pyrosequencing											x							x				x	x		[49]
Zemanick ET	2013	37 CF	Sputum	Conventional culture 16S rRNA pyrosequencing		x	x			x	x		x	x		x	x			x	x	x		x	x		x	x	[35]
Delhaes L	2012	8 CF	Sputum	Conventional culture 16S rRNA pyrosequencing									x		x					x	x	x					x	x	[39]
Fodor AA	2012	23 CF	Sputum	16S rRNA pyrosequencing					x												x	x		x			x	x	[37]
Stressmann FA **	2012	14 CF	Sputum	Conventional culture analysis T-RFLP																		x			x	x	x		[44]
Zhao J	2012	6 CF	Sputum	16 s rRNA pyrosequencing									x								x	x		x			x	x	[41]
Guss AM	2011	4 CF	Sputum	16 s rRNA pyrosequencing		x		x					x							x	x			x			x	x	[50]
Sibley CD	2011	6 CF	Sputum	Conventional culture T-RFLP 16S rRNA pyrosequencing	x	x		x	x						x						x	x			x	x	x	x	[51]
Cox MJ***	2010	63 CF	Sputum Throat swab	Microarray																		x			x				[52]
J Harris JK ****	2007	28 CF 14 healthy	Bronchoalveolar lavage fluid	DGGE/TGGE											x							x			x		x		[53]
Rogers GB *****	2004	34 CF	Sputum	T-RFLP								x						x	x	x	x	x	x						[54]

*Stenotrophomonas maltophilia, Streptococcus agalactiae, Haemophilus influenzae, Pseudomonas aeruginosa.

**P. aeruginosa, *Stenotrophomonas maltophili*’, *Staphylococcus aureus*, *Streptococcus* Group F.

***S. aureus, *P. aeruginosa.*

****S. aureus, S. maltophilia, P. aeruginosa, Streptococcus mitis, *H. influenza.*

*****P. aeruginosa, *Porphyromonas endodontalis, P. gingivalis, Prevotella denticola, P. melaninogenica, P. nigrescens, P. veroralis, P. intermedia*, *P. loescheii, P. salivae, P. buccae, P. oris, Craurococcus roseus, Rhizobium loti, Ochrobactrum anthropi, Peptostreptococcus anaerobiu*s.

Asterisks indicate studies in which mentioned species were identified.

### The microbiome in chronic rhinosinusitis

Specimen collection is one of the most important steps in the analysis of remote areas such as the sinuses. An appropriate collection of the samples is the first step to perform a meaningful, high quality analysis. It must not be biased by interference from the nares. Specimen can be tissue, nasal secretions [55] or material sampled by a swab. The use of an endoscope for the sampling during sinus surgery is advisable [17,18,56], although simple swabs are often used [57] in both healthy and diseased patients [18]. Samples can be collected from various anatomical locations in the nose such as the inferior turbinate [55], the middle-meatus [56], the ethmoidal sinuses, the sphenoid [18], and the anterior nasal cavity [58]. Mucosal surfaces of the lateral, central, and medial portions of the maxillary sinus are also collected from locations in the nose [17]. The use of middle-meatus swabs for DNA-based bacterial assays is appropriate for the detection of multiple bacterial species, including anaerobes, which may be undetected when swabs are used solely for culture. Based on available cultivation-based studies, the microbiology of the middle meatus correlates well with pathogenic organism of CRS, whereas swabs of the nares would not be appropriate as a replacement of middle meatus swabs in investigations of CRS pathogen [59]. Swabs should not be contaminated by the microorganism of the nares during insertion/retraction from the middle meatus or sinuses [56]. To avoid contamination by the nasal vestibule, researchers often use appropriate protective devices such as a sterilized Killian nasal speculum with long leaves [58].

Before the era of culture-independent methods, conventional cultures have implicated *Staphylococcus aureus* and coagulase-negative *Staphylococcus* as principal pathogens in chronic rhinosinusitis (CRS) [60]. The development of culture-independent molecular techniques allowed the detection of more bacteria [60] and revealed greater biodiversity than conventional culture [56]. Thus, the etiology of CRS may be polymicrobial [55] and the role of anaerobe bacteria may be more prominent than presumed; however, it is likely that the bacteria detected by culture-dependent techniques still are of clinical relevance [60].

Using comparative microbiome profiling in a cohort of a small number of not further defined CRS patients and healthy subjects, it was proposed that the sinus microbiota of CRS patients exhibit significantly reduced bacterial diversity compared to those of healthy controls. Abreu et al., found a depletion of multiple phylogenetically distinct lactic acid bacteria coincident with an increase in the relative abundance of a single species, *Corynebacterium tuberculostearicum* [17]. These microbe caused goblet cell hyperplasia and mucin hypersecretion in a murine model of sinus infection. In this model, *Lactobacillus sakei* represented a potentially protective species [17]. However, the finding of this single species has not been confirmed by others [55,56] (see Table 2). In a larger study, *Staphylococcus aureus* and *Propionibacterium acnes* were the most common organisms in CRS (mostly CRSwNP) and controls, respectively [18]. Recently, the investigators detected *Staphylococcus aureus*, *Staphylococcus epidermidis* and *Propionibacterium acnes* as the most prevalent and abundant microorganisms in healthy sinuses [61]

**Table 2:** **Summary of chronic rhinosinusitis microbiota studies; type of sample, technique used and genus identified**

**Author**	**Year**	***n***	**Sample**	**Techinque**	***Aureobacterium***	***Alicycliphilus***	***Burkholderia***	***Carnobacterium***	***Citrobacter***	***Cloacibacterium***	***Corynebacterium***	***Curtobacterium***	***Cyanobacterium***	***Diaphorobacter***	***Enterobacter***	***Enterococcus***	***Fusobacterium***	***Haemophilus***	***Helicobacter***	***Lachnospira***	***Lactobacillus***	***Micrococcus***	***Moraxella***	***Mycobacterium***	***Neisseria***	***Pediococcus***	***Peptoniphilus***	***Propionibacterium***	***Pseudomonas***	***Rhodococcus***	***Staphylococcus***	***Stenotrophomonas***	***Streptococcus***	**Ref.**
Ramakrishnan VR et al.*	2013	28 healthy	Middle meatus swab	qPCR 16S rRNA pyrosequencing							x				x		x	x					x		x			x			x	x	x	[61]
Aurora R et al.	2013	30 CRS 12 healthy	Superior middle meatus lavage	16S rRNA pyrosequencing		x				x	x	x	x																					[62]
Boase S et al.**	2013	38 CRS 6 healthy	Mucus Middle meatus swab	Conventional culture Ibis T5000																								x			x			[18]
Abreu NA et al.	2012	10 CRS 10 healthy	Brush samples of mucosal surfaces of the lateral, central, and medial portions of the maxillary sinus	PhyloChip analysis			x	x			x					x			x	x	x			x		x			x					[17]
Feazel LM et al.**	2012	15 CRS 5 healthy	Middle meatus swabs	Conventional culture 16S rRNA pyrosequences							x																	x			x			[56]
Stressmann FA et al.***	2011	43 CRS.	Polyp and inferior turbinate tissue. Mucin (if present)	16S rRNA pyrosequencing T-RFLP					x									x										x	x		x		x	[55]
Frank DN et al.****	2010	26 S. aureus carriers 16 non-carriers 5 healthy	Nostril swabs	16S rRNA pyrosequencing							x				x	x												x			x			[63]
Stephenson MF et al.*****	2010	18 CRS 9 healthy	Mucosal Biopsy	16 s rRNA pyrosequencing										x													x				x			[60]
Healy DY et al.******	2008	11 CRS 3 healthy	Mucosa samples	FISH DNA probes *Haemophilus influenzae Streptococcus pneumophilia Staphylococcus aureus Pseudomonas aeruginosa*														x											x		x		x	[64]
Sanderson AR et al.*******	2006	18 CRS 5 healthy	Biopsies of the sinus mucosa	FISH DNA probes *Haemophilus influenzae Streptococcus pneumophilia Staphylococcus aureus Pseudomonas aeruginosa*														x											x		x		x	[65]
Lina G et al.********	2003	216 healthy	Nasal vestibule swabs	Conventional culture							x											x									x		x	[66]
Rasmussen TT et al.*********	2000	10 healthy	Nasal washes	Conventional culture Capillary sequencing	x						x																			x	x			[67]

*S. aureus, P. acnes.

**P. aeruginosa.

***S. aureus, S. epidermidis, Propionibacterium acnes.

****S. aureus, S. coagulase-negative, S. anaerobic species.

*****H. influenza, Streptococcus pneumophilia, S. aureus, P. aeruginosa.

******Staphylococcus aureus, S. non-aureus, S. epidermidis, S. capitis, S. haemolyticus, S. warneri, S. hominis, S. lugdunensis, S. cohnii subsp. cohnii, S. auricularis.

*******Haemophilus influenza, S. pneumoniae, S. aureus, Pseudomona aeruginosa.

********Staphylococcus epidermis. S. capitis, S. hominis. S. haemolyticus, S. lugdunensis, S. warneri.

Asterisks indicate studies in which mentioned species were identified.

.

Using culture-independent (qPCR and 16S rRNA gene sequencing) methodologies for pathogen identification in chronic rhinosinusitis patients, among 57,407 pyrosequences were generated. The most prevalent ones were from coagulase-negative staphylococci (100%), 21/21 specimens, *Corynebacterium spp* (not specifically *Corynebacterium tuberculostearicum*) (85.7%) 18/21, *P. acnes* (76.2%), 16/21, and *Staphylococcus aureus* (66.7%) 14/21. Although these authors found significantly different distributions of 16S rRNA sequences recovered from CRS vs. non-CRS cases, neither richness nor evenness indices showed statistically significant differences [56]. In another approach using 16S rRNA gene clone sequencing in a terminal restriction fragment length polymorphism (T-RFLP) analysis, the bacteria present in 70 clinical samples from 43 CRS patients undergoing endoscopic sinus surgery were characterized; a total of 48 separate bands were detected. Species belonging to 34 genera were identified as present by clone sequence analysis. Of the species detected, those within the genera *Pseudomonas, Citrobacter, Haemophilus, Propionibacterium, Staphylococcus*, and *Streptococcus* were found numerically dominant, with *Pseudomonas aeruginosa* being the most frequently detected species [55]. Another prospective study collected mucosal biopsies from 18 patients undergoing endoscopic sinus surgery for CRS and 9 control patients with healthy sinuses (indication: pituitary adenomas) compared swab culture with bTEFAP (bacterial tag-encoded FLX amplicon pyrosequencing). Standard cultures mainly showed *Staphylococcus aureus* and Coagulase-negative *Staphylococcus aureus*, whereas the molecular analysis identified up to 20 predominant organisms per sample. *Staphylococcus aureus* was nevertheless detected in about 50%; moreover, they disclosed anaerobic species with so far unknown impact in CRS, *Diaphorobacter* and *Peptoniphilus*. Interestingly, *Diaphorobacter* is described as a strong biofilm creator [55,60].

Table 2 provides a summary of previous studies related to the microbiome in chronic rhinosinusitis, including sample size, type of sample, technique used and genus found.

Comparisons of molecular analyses suggest that the detection of microorganisms by Fluorescence *in-situ* hybridization (FISH) and culture-dependent techniques is related to the abundance of an organism, furthermore, cultivation tends to give advantage to rapidly growing bacteria [18]. The investigators employed conventional cultivation, molecular diagnostics and FISH to detect *Staphylococcus aureus* as a standard. They found that FISH analysis had a sensitivity of 78% with a specificity of 93% compared to the molecular technique [18]. Evidence from high-sensitivity techniques demonstrates that the healthy sinus is clearly not sterile [18], but shows high diversity of the resident microbiota [17]. The nasal microbiota of healthy subjects mainly consist of members of the phylum *Actinobacteria* (e.g., *Propionibacterium spp*. and *Corynebacterium spp*.), whereas the phyla *Firmicutes* (e.g., *Staphylococcus spp.*) and *Proteobacteria* (e.g. *Enterobacter spp*) are less frequent [55,56,60,63]. It appears that the prevalence and abundance of organisms is critical in determining healthy conditions [18].

Thus, similar to CF, findings in CRS have pointed out that the microbiome is unique for each individual patient [42-44] and the community of microbes is diversified [10]. As a general principle, a decreasing bacterial diversity is correlated with disease severity in CF [37-39,42,44], whereas CRS patients were characterized by an altered microbial composition and greater abundance of *Staphylococcus aureus* [56]. There was no single common microbiota profile among patients with similar clinical conditions in the studies performed so far, although *Staphylococcus aureus* was prominent in most studies [10,11,68]. Thus, there is a clear need for larger series of well-defined patients sampled and investigated in an optimal way, also avoiding the interference of recently applied antibiotics, to establish the correlation between microbe and CRS disease.

### Limitations of the current studies

Airway microbiome studies revealed several critical factors, which also may impact CRS studies. First of all, the inclusion of well-defined patients, using pheno- and potentially endotypes of upper airway disease [68-71], and matched controls in meaningful numbers is necessary to draw supportable conclusions. Furthermore, recent antibiotic treatment within 1 month [44] prior to collection could significantly reduce the diversity of the microbiome in samples [42,43,56], and contamination by bacteria from other organs such as the skin should be taken into account [9,27]. Factors which may perturb the collection or evaluation procedures are contaminating host DNA [40] or RNA, the existence of viruses such as bacteriophages in the samples, which may impact on the number and genes of microbes [72], and technical issues such as extraction methods (e.g. modified lysostaphin-lysozyme method to enhance staphylococcus DNA extraction) [41].

Currently, most of the publications in human microbiome studies have spotlighted sequencing of 16S rRNA in the identification of bacteria. Their results may misjudge the level of diversity and microbial composition by amplification of chimera and pseudogenes and/or inappropriate primer selection. Metagenomic shotgun sequencing may avoid these problems by omitting amplification and allows to detect gene contents of complex microbiota and to compare functional gene contents between samples, but still may have limitations as discussed above and in low-microbial burden samples. However, researchers are now increasingly employing novel techniques to study the human microbiome [25].

### Conclusion and perspective of nasal microbiome studies

The new molecular techniques enhance our chance to identify new bacteria within the nose and nasal cavities; as the pivotal host functions evolved under high microbial pressure, they will show a very complex network of microbes and thus microbe-host interactions [41]. On the host side, specific pheno- and endotypes of CRS have been described characterized by an imbalance of Th1 and Th2 function [71]. In CRSwNP patients, *Staphylococcus aureus* has been identified to unfold impact on the mucosal immune functions [10,68,70]. The relationship between the microbiome and mucosal immunity may be bidirectional, with pressure coming from the bacteria and inadequate defense from the host [70]. Research on how specific bacteria impact on the immune response of nasal and sinus mucosa may shed new light on the pathophysiology of CRS and may result in new strategies for its treatment.

The manipulation of microbiota or the introduction of specifically healthy microbiota may prove to be useful for the treatment of inflammatory disease [73]. *Staphylococcus aureus* and *Pseudomonas aeruginosa* are principal offenders in the development of persistent severe airway disease in CRS and CF patients. As bacterial resistance complicates the efficacy of antibiotics, the use of probiotic bacteria as colonizers and antimicrobial agents that may inhibit the growth of pathogenic bacteria awaits further development.

## Abbreviations

CF: Cystic fibrosis
CRS: Chronic rhinosinusitis
CRSwNP: Chronic rhinosinusitis with nasal polyps
DNA: Deoxyribonucleic acid
RNA: Ribonucleic acid
rRNA: Ribosomal ribonucleic acid
bp: Base pairs
FISH: Fluorescence in situ hybridization
PCR: Polymerase chain reaction
qPCR: Quantitative polymerase chain reaction
T-RFLP: Terminal restriction fragment length polymorphism
bTEFAP: Bacterial tag-encoded FLX amplicon pyrosequencing
